# The Limb Kinetics of Goat Walking on the Slope with Different Angles

**DOI:** 10.3390/biomimetics7040220

**Published:** 2022-11-30

**Authors:** Weijun Tian, Jinhua Zhang, Kuiyue Zhou, Zhirui Wang, Ruina Dang, Lei Jiang, Ju Wang, Qian Cong

**Affiliations:** 1 Key Laboratory of Bionic Engineering (Ministry of Education, China), Jilin University, Changchun 130022, China; 2 North-Vehicle Research, Fengtai District, Beijing 100072, China; 3 Pujiang Agricultural and Rural Bureau, Chengdu 322200, China

**Keywords:** boer goat, slope, kinetics, center of mass

## Abstract

The study aimed to assess the gait adjustment techniques of limbs on different slopes and investigate the relationship between forelimb and hindlimb kinetics and the center of mass (COM) during the uphill movement of a specific Boer goat using a pressure-sensitive walkway (PSW). During the uphill and downhill movements at a comfortable walking speed, we measured the ground reaction force (GRF) of the forelimbs and hindlimbs on the slope, the change in the included angle of the propulsive force direction of the forelimbs and hindlimbs, and the impulse relationship between GRF and propulsive force. According to the study, since the forelimbs of the goat were nearer the COM, they were primarily adjusted during the movement on the slope. By lowering the initial included angle of the propulsive force and the angle variation range, the forelimbs and hindlimbs could walk steadily. The forelimbs and hindlimbs exhibited completely different adjustment strategies during uphill and downhill movements. In particular, the forelimbs performed braking and the hindlimbs performed driving. In addition, we discovered that the goat altered its adjustment strategy when climbing the steep slope. All findings of this study indicate the need to understand the gait adjustment mode of the Boer goat during movement on the slope to thoroughly comprehend the driving strategy of quadrupeds with the ability to walk on specialized terrains.

## 1. Introduction

The legged robot has received much attention in the past few years because of its excellent performance in complex environments [1,2,3]. Improvement of stability and gait control of the legged robot is an important research issue; quadruped robots have better mechanical load capability and stability than biped robots. Quadruped robots can not only walk on uneven ground and complex topography by static walking but also at high speed by dynamic walking, and the development of bionics has provided new research ideas for the improvement of movement ability for quadruped robots [4,5]. In the wild, quadrupeds move across various challenging and unique terrains through the alternating, periodic actions of their limbs [6]. Coordination between the limbs results in different gait changes [7]. Animals produce gait motions through the operation of body kinetics by the movement system according to their surroundings [8]. How and why animals employ particular gaits can be understood by observing how they choose to move in various contexts [9,10]. The excellent motion characteristics of tetrapods provide bionic ideas for the design of quadruped robots. A sufficient theoretical basis and great bionic blueprint can be provided for the design of stability and topography adaptability for high-performance quadruped robots by collecting and analyzing kinematic data of tetrapods and studying their gait and motion characteristics under some complex topography.

Gait analysis can help us comprehend the motions of animals more clearly [11,12]. Gaits can be analyzed in various ways. Higher standards are needed for researchers to perform a subjective visual analysis, and different observers have different perspectives [13,14]. Veterinarians have long utilized computer-based image analysis techniques to analyze the marked locations after capturing pictures with a camera [15,16,17]. More and more objective evaluations have been made for the gait analysis of medium-sized quadrupeds, such as horses and cattle [18,19,20,21,22,23]. However, acceptable standards for thoroughly examining the gaits of goats are lacking [24]. Pressure-sensitive walkways (PSWs) and force plates are frequently used in the objective kinetics collection system of quadrupeds [25]. Pressure-sensitive walkways can only collect pressure in the vertical orientation, while force plates can collect pressure in many directions, providing more complete data [26]. However, applying PSWs can greatly lower testing difficulties for quadrupeds that are challenging to train [27].

Recently, numerous gait studies on quadrupeds have been conducted, primarily focusing on horses and dogs with high domestication levels. However, as objective methods for collecting data have become more popular, studies on cats are also progressively expanding [25,28]. Numerous temporo-spatial and kinetic gait metrics of limbs were gathered from quadrupeds in earlier research [18,19,25,27,28,29,30]. For several breeds of the same species, noticeable variations existed in the forelimb and hindlimb kinetics [31,32]. The differences between the sexes, in contrast, were far less pronounced [33]. There was a noticeable difference between the forelimbs and hindlimbs but not between the left and right paired limbs [26,34,35]. Perhaps because the gaits of goats need to be adequately taught before gait analyses due to some objective characteristics, such as gregariousness, related studies are few [24,35,36].

Most previous studies on quadrupeds were conducted on level ground, while studies on unique terrains, such as slopes, are few. Unlike the pressure distribution and tactical roles of the forelimbs and hindlimbs of quadrupeds on level ground, those on slopes are significantly altered [20]. In addition, the position of the center of mass (COM) also shifts in slope situations, significantly affecting the kinetics of the limbs [37]. Further knowledge of some characteristics of the gaits of goats is needed because of their capacity to traverse inclined terrains [35]. The ground reaction force (GRF) and the vertical force distribution of the hoof pressure of goats on slopes have been studied, but analyses and research on limb gaits are lacking [38,39]. Studies on the gait and kinetic characteristics of animals have continued to develop. However, it is still challenging to comprehend how animals modify their gaits and how their gaits differ depending on terrains [40]. Many concave-convex terrains are overlayed by slopes with different gradients in conventional complex terrains. Therefore, studying the kinematics characteristics of quadruped robots on slopes is crucial to realize stable motion under complex terrains.

This study aimed to investigate the limb kinematics and COM of a Boer goat when it is walking on a slope. The kinetic link between the slope angle and the COM-related propulsive force vector provided by the limbs was examined while the Boer goat was trained to walk uphill and downhill. Our findings will contribute to a deeper understanding of the limb modulation approach that enables goats to walk on slopes. We hypothesized that when the slope angle increased, the forelimbs and hindlimbs might adopt a different driving style from that used on flat ground. The research will provide methods for the leg design and gait control of quadruped robots.

## 2. Materials and Methods

### 2.1. Animal

The animal care and use committee of Jilin University gave its approval to all procedures in this study. A sizable number of Boer goats were not enlisted for this study due to safety issues in conducting the experiment in the laboratory. We used an 8-month-old 44.3-kg male Boer goat bought from a certified farm in Changchun. It was carefully selected from healthy goats without a history of bone illness, and for the time being, the influence of variations in goat body size was not considered. We selected a well-proportioned goat with great athletic ability that had no injuries or lameness impeding its regular gaits after a long visual examination of the movement states of the goats as they moved on flat terrains. We used the sample collection strategy similar to that used by Lewinson et al. in the description experiment of goat climbing kinetics, in which the relatively low differentiation degree may represent some of the population features of the participant in this experiment [41]. We conducted several tests under the guidance of relevant experts to decrease experimental errors and increase the reliability of the findings.

### 2.2. Experimental Scheme Design

**Figure 1:**
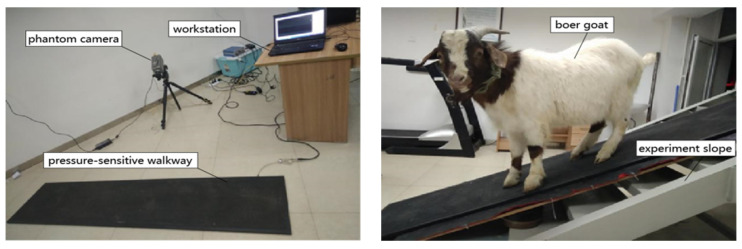
Experimental equipment. The walking data of the goat on the slope are collected by the Phantom camera control system and the PSW and analyzed by the workstation.

The experiment was conducted in the Biomechanics Laboratory of Jilin University. The experimental equipment comprised a slope (self-built, with a size of 2.5 × 1.4 m), of which the slope angle could be adjusted manually and increased linearly from 10° to 30° at intervals of 10°. We took pictures and videos of the slope region with a Phantom high-speed camera and evaluated the motion data of the plantar markers of forelimbs and hindlimbs using a Phantom camera control system at a sampling frequency of 20 Hz. To gather kinetic GRF data on the slope system, a 2096 × 469 mm PSW in which a 7.62 × 5.08 mm sensor was implanted with a sampling frequency of 120 Hz was put in the middle of the slope (in Figure 1). The PSW was synchronized with the motion capture system after being balanced, calibrated, and synchronized according to the manufacturer’s specifications.

### 2.3. Data Collection

The Boer goat was allowed to explore the room on the PSW and accustom it to the surroundings before the formal experiment. The goat could adjust to walking on the slope with different angles due to the integrated slope device. To ensure that the goat walked at a comfortable pace and the soles of its feet were in complete contact with the PSW for many times prior to data collection, the goat was fed prior to each experiment to induce it to pass through the slope with a smooth and harmonious gait. When the goat rested throughout the experimental time, the three slope angles and the uphill and downhill test sequences were randomly grouped to somewhat ensure the validity of the findings. When the goat walked at a comfortable walking pace, its head was in the middle, and its limbs made full contact with the PSW, the experimental findings were valid. The experimental results were disregarded if the pace of the goat significantly varied, there was visible resistance and reluctance, and the limbs made only partial touch with the PSW. The gait of the goat was captured by the camera throughout the experiment, and the same kind of tests were conducted on the same day. The goat either completed five successful tests or walked at least ten times in each walking scenario. The missing data points in the first and last three frames of data were fixed using spline interpolation.

### 2.4. Data Analysis and Outcome Parameters

**Figure 2:**
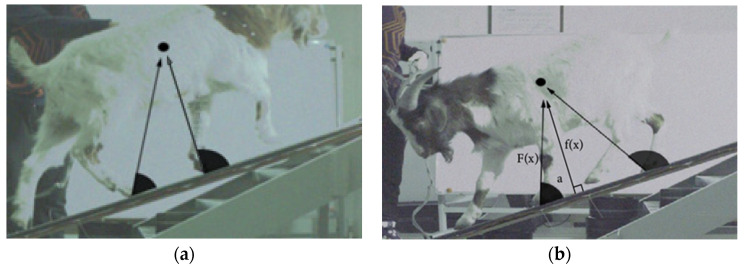
Graphical representation of the angle. The black point is the COM, and the included angle is between the propulsive force F(x) and the upward direction along the slope: (**a**) uphill; and (**b**) downhill.

The supporting forelimbs and hindlimbs of the goat produce a propulsive force when it walks on a slope, which was utilized to measure changes in the angle between the propulsive force vector and the slope. We defined the propulsive force as the force generated by the feet of forelimbs and hindlimbs in the direction along COM. As illustrated in Figure 2, the sole and COM are manually linked as the propulsive force vector by phantom high-speed camera processing software for the camera video data taken while walking. We drew on Lee’s estimation of dog COM under the slope condition for the COM estimation, locking the COM position between the shoulder and hip [37]. By observing the reaction loads on the support surfaces of the forelimbs and hindlimbs when resting on the slope, it was noted that the forelimbs support 60.3% of its weight, and the hindlimbs support 39.7%. Therefore, we simplified the data that forelimbs account for about 60%, and hindlimbs account for 40%. The COM position should be at 40% of the trunk, biased toward the forelimbs. The angle change between the left forelimb and the right hindlimb was investigated in this research because there is no difference between the paired limbs of the goat [24]. The propulsive force can be expressed as the following equation:

F(x) = f(x)/sin(a),(1)

where F(x) is the propulsive force, f(x) is the vertical pressure, and sin(a) is the included angle between F(x) and the slope. Impulse will be generated under the action of force during the motion of goats. Impulse is the integral of force over time, and is calculated by area. Impulse analysis was performed based on the area calculation of the force-time function curve.

### 2.5. Statistical Analysis

To compare data under various slope circumstances, a statistical analysis was performed using a one-way analysis of variance by Statistical Package for Social Science with post hoc Bonferroni correction. A statistically significant difference is observed when *p* < 0.05.

## 3. Results

### 3.1. GRF-Time Curve

**Figure 3:**
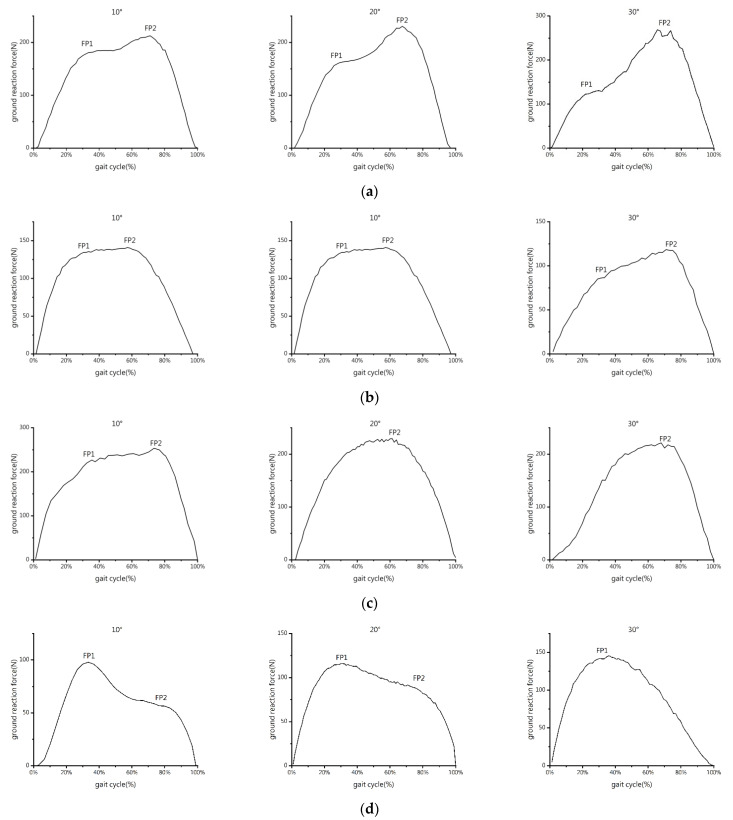
GRF-time curves of different limbs of the goat under different slope conditions. The FP1 is the peak value of landing, and the FP2 is the peak value of push-off: (**a**) forelimb, uphill; (**b**) hindlimb, uphill; (**c**) forelimb, downhill; and (**d**) hindlimb, downhill.

The force perpendicular to the surface of the slope was the GRF of the left forelimb and right hindlimb of the goat. We used the pressure test system to capture the GRF-time curve data of the forelimb and hindlimb while the goat was walking on the constructed slope (Figure 3). The former segment of the GRF-time curve, which corresponds to the landing peak value (FP1), and the latter segment, which corresponds to the push-off peak value (FP2), were both examined [23]. FP1 and FP2 are the two peaks of the reaction force f(x) curve measured by PSW perpendicular to the support surface (slope).

As shown in Figure 3, the GRF-time curve of the forelimb displayed two local maxima during uphill, and the FP1 is smaller than the FP2 at any slope angle. With an increase in slope angle, the FP1 lowers, and the FP2 rises. When the slope angle is 10°, the GRF-time curves of the FP1 and FP2 of the hindlimb are comparable. The FP1 is greater than the FP2, and both reach their maximum values when the slope angle is 20°. At a slope angle of 30°, the FP1 is smaller than the FP2, and both are at their lowest. With an increase in slope angle, the location of the FP1 of the forelimb gradually moves from 30% to 20%, whereas the FP2 essentially stabilizes at 70%. The position of the FP2 of the hindlimb moves from 60% to 75%, and the FP1 essentially stabilizes at 30%.

The GRF-time curve of the forelimb only has a double peak at a slope angle of 10° during downhill, the FP1 is smaller than the FP2, and both are the highest peaks for each slope. Only the FP2 is still present at slope angles of 20° and 30°, and the FP2 at the slope angle of 20° is close to that at 30°. The FP1 and FP2 of the hindlimb increase with the slope angle throughout the downhill phase. Double peaks at slope angles of 10° and 20° are visible in the hindlimb GRF-time curve, with the FP1 being greater than the FP2. When the slope angle approaches 30°, only the FP1 still exists and reaches its maximum. The location of the FP2 of the forelimb is advanced from 75% to 65% and then delayed to 70% as the slope angle increases. The double peak positions of the hindlimb remain consistent between 30% and 80%.

The GRF of the forelimbs of the goat was determined to account for around 60% of the total by calculating the average value of the peak vertical forces of the forelimb and hindlimb when the goat walks up and down the slope. Therefore, in the following study, the COM of the goat during walking on the slope is defined as the front 40% of the body.

### 3.2. Included Angle between the Propulsive Force and Slope

**Figure 4:**
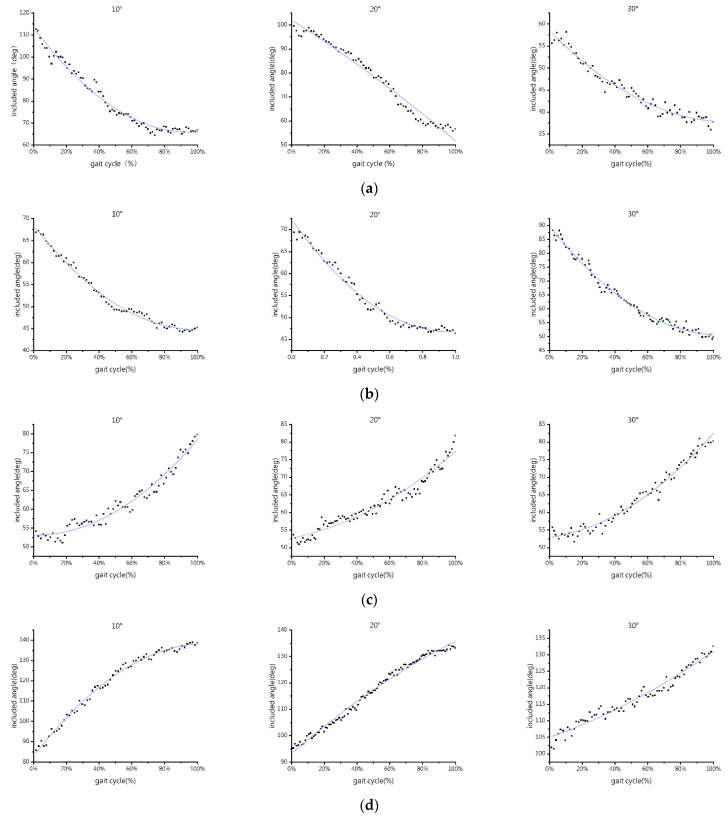
Included angle-time point plots of different limbs of the goat under different slope conditions: (**a**) forelimb, uphill; (**b**) hindlimb, uphill; (**c**) forelimb, downhill; and (**d**) hindlimb, downhill. The blue line is the second-order fitting to describe the point tendency.

Figure 4 depicts the information on the angle between the slope and the propulsive force vector.

As shown in Figure 4, the angles between the propulsive force and the slope of the forelimb and hindlimb both reduce as the slope angle increases during uphill. The initial included angles of the forelimb gradually reduce. The initial included angles of the forelimb at slope angles of 10° and 20° are 115° and 100°, respectively, both greater than 90°, and the change range of included angles is close to 50°. The initial included angle of the forelimb is 55°, suddenly smaller than 90°, at a slope angle of 30°, with a change range reducing to 20°. The initial included angles of the hindlimb are all below 90° and subsequently rise. The initial included angles of the hindlimb are around 70° at slope angles of 10° and 20°, and the change range is about 25°. At a slope angle of 30°, the initial included angle of the hindlimb is approximately 88°, with a change range rising to 40°.

The angles between the propulsive force and the slope of the forelimb and hindlimb increase with the slope angle during downhill. They are all around 55°, smaller than 90°. There is little change in the initial included angle of the forelimb. At a slope angle of 10°, the change range of the included angle of the forelimb is 50°, while at slope angles of 20° and 30°, the change ranges are only around 27.5°. The initial included angle of the hindlimb is 85°, falling below 90°, at a slope angle of 10°, and the change range is close to 50°. At slope angles of 20° and 30°, the change ranges of the hindlimb are 40° and 30°, respectively, with the initial included angles of 95° and 102°, which are also greater than 90°.

The direction of the propulsive force produced by the forelimbs and hindlimbs is determined by the angles between their respective propulsive force and the slope. The component of the propulsive force of the forelimb along the slope is opposite to the forward direction of the goat when the included angle is larger than 90°, leading to a braking action. The slope components of the propulsive force of the forelimb and hindlimb are consistent with the forward direction when the included angle is smaller than 90°, and the propulsive force has the driving effect. The connection between angle and propulsive force during downhill is completely opposite to that during uphill. Therefore, the driving effect of the entire operation is provided by the propulsive force of the hindlimb. When the slope angle is minimal, the forelimb acts as a brake, and when the slope angle is great, it acts as a driver [37].

### 3.3. Propulsive Force-Time Curve, GRF-Time Curve and Impulse

**Figure 5:**
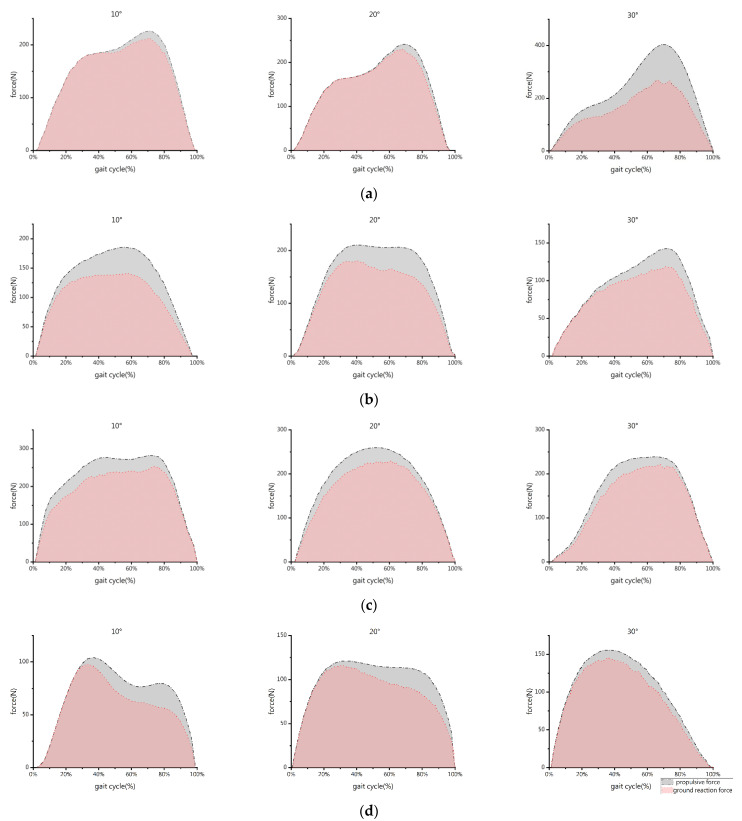
Impulse areas of different limbs of the goat under different slope conditions: (**a**) forelimb, uphill; (**b**) hindlimb, uphill; (**c**) forelimb, downhill; and (**d**) hindlimb, downhill. Red indicates the impulse of the GRF, and gray refers to the impulse of the propulsive force.

Figure 5 shows the comparative curves of propulsive force-time and GRF-time for the forelimb and hindlimb. The impulse values of the propulsive force and GRF are listed in Table 1.

Overall, the thrust time curve and the pressure time curve have the same law. During uphill, the FP2 difference between the forelimb and hindlimb is greater than the FP1 difference. The impulse values of the propulsive force of the forelimb and hindlimb are almost equal to 140 at slope angles of 10° and 20°. At a slope angle of 30°, the forelimb has the maximum impulse value of the propulsive of 200, and the hindlimb has the lowest impulse value of 90. At high slope angles, the impulse of the forelimb and hindlimb dramatically increase.

In the downhill process, for the forelimb, the FP1 difference is marginally greater than the FP2 difference. However, for the hindlimb, the situation is the opposite. The impulse value of the propulsive force of the forelimb is about 200 at a slope angle of 10° and close to 160 at 20° and 30°. The impulse value of the propulsive force of the hindlimb is at least 70 at 10° and almost 100 at 20° and 30°. The combined impulse of the forelimb and hindlimb during downhill is stronger than that during uphill. The impulse values of the forelimb are typically higher than those of the hindlimb.

## 4. Discussion

Goats are ruminants that enjoy climbing to great heights, excel at jumping, and can traverse challenging terrains like cliffs and steep slopes. Sufficient comprehension of the relationship between the propulsive force of the limbs when a goat moves over sloping ground is lacking. This research aimed to study the connection between the limb kinetics and propulsive force of the Boer goat to better understand how quadrupeds regulate their gaits when traversing unusual terrain.

In the gait analysis of the goat, we used the PSW to record the pressure data of the forelimb and hindlimb when the goat was walking on the slope at a comfortable walking pace. Then, the weight ratio of the forelimb and hindlimb was used to calculate the location of the COM during the slope movement [21]. The included angles between the propulsive force and slope and the impulse values of the propulsive force were then gathered and examined after connecting the sole of the forelimb and hindlimb with the COM as the propulsive force direction. This approach is different from that used in early studies on goat gaits [24,35,36]. On flat ground, the proportions of the forelimb and hindlimb vary greatly among quadrupeds, but the proportions of the forelimb are all at least 60% [30,35,36]. Even if a quadruped drops dramatically on a slope, the proportion of the forelimb is still higher than that of the hindlimb [20,24]. Therefore, during walking on a slope, the COM position is closer to the forelimb, which is consistent with the findings of this study. It suggests that during walking on slopes, the role of forelimbs may be more prominent.

Animals must land first and then push off to make steps. Therefore, there are two peaks called the landing peak and the push-off peak, respectively, at the former and latter segments in the force-time curve. On flat ground, the two peaks essentially show up in the former and latter quarters of the gait cycle [23]. The findings of this study are different, and the relationship between the walking speed and limb preference is unclear [21,22]. These anomalies demonstrate that, with an increase in slope, the forelimbs must resist the downhill component of the gradually increasing body gravity along the slope during uphill. Therefore, to increase the time spent on the slope and the walking stability of the forelimb and hindlimb when they walk alternately, the landing time of the hindlimb is advanced, and its push-off time is staggered. During downhill, the forelimb only needs to push off to braking, while the hindlimb only needs to land to achieve driving. At a slope angle of 30° during uphill, the hindlimb inverts their landing peak value and push-off peak value to add some driving force. The other peak patterns of the forelimb and hindlimb agreed with those in early research [22,23].

According to early research, when dogs walk on slopes, their limbs serve as levers and props to vary their limb angles in response to variations in slope [37]. Similar conclusions were obtained in this paper. However, the shift in the hindlimbs is not immediately apparent, and the change range of the angles of the forelimbs reduces as the slope increases. The angle results also indicated that the COM was deflected to the forelimbs, and the forelimbs were more sensitive to slope changes than the hindlimbs. In order to lower the COM and preserve the stability of the COM to sustain stable walking on the slope, the goat primarily modified its forelimbs by reducing the initial included angle and minimizing the change range. The findings also demonstrate that, during uphill and downhill, the forelimbs serve as brakes, and the hindlimbs as drivers. Contrary to early experiments, the forelimbs were converted to drive to improve the capacity of goats to climb steep slopes [42]. The variance may be due to the various testing paces and goat breeds.

According to the impulse results of the propulsive force presented in this study, when the goat was climbing a steep slope, the forelimbs pressed the ground firmly, while the hindlimbs reduced some of the driving effort to conserve physical energy. The primary weight of the goat was transferred to its forelimbs during downhill. Because of the gravity, the forelimbs greatly slipped at a slope angle of 30°, and the hindlimbs only needed to give a modest propulsive force similar to that at a slope angle of 20° to move the goat downhill.

The goat pushed backward with its forelimb and hindlimb simultaneously to decrease the initial included angles of the forelimb and hindlimb and enhance the impulse of the propulsive force to produce enough driving force when moving uphill on a steep slope. The stability was maintained by reducing the angle change range during uphill. As expected, the hindlimb would adapt to a walking position similar to that of the forelimb and reduce some of the driving force to conserve energy because the forelimb could produce enough driving force.

The forelimb and hindlimb would adopt a forward-backward posture, and the change range was also decreased to preserve stability, in order to generate certain friction force to resist the gravity component during downhill. While the hindlimb would continuously to generate driving force similar to that in the previous stage and give certain friction force by altering the angle to stabilize walking, the forelimb could no longer supply enough braking force when the slope was steep.

These results showed that when walking on the slope, the goat could use specific strategies to adjust the kinetics of the forelimbs and hindlimbs to conserve energy while maintaining efficiency and stability [42]. The forelimbs were significantly impacted by the change in slope, and the hindlimbs could be adjusted appropriately in response to the forelimbs. In addition, we discovered that the initial included angle of the forelimb was around 55° during uphill on the steep slope and downhill on each slope. This finding may be related to the physiological structure of the goat forelimbs, which will be further explored in the future.

Through the interaction between the body parts and the environment, animals can drive the internal and external passive degrees of freedom to move in the physical environment. For example, fish swim in the water by passively combining their soft bodies with the whirlpool of the surrounding water. Moths passively twist their wings to generate the lift for hovering flight [43]. The dexterous arms of an octopus can have powerful driving forces through the complex fluid dynamics with the surrounding current generated by the hybrid periodic motion with alternating speed [44]. Snails and other mollusks show the characteristics of chaotic behavior in their motion patterns [45]. When cats conduct obstacle-avoidance walking, they will have a hybrid motion mode that changes the direction of the stride for the same duration or reduces the stride for the same duration [46]. Similarly, the research on the complex motion of similar quadruped animals and quadruped robots can be simplified to the research on biped models and robots in some cases. The quadruped robot can be decomposed into coupled biped robots, which can be applied to the quadruped robot by recombining the biped gait [47,48]. Therefore, the research on the bipedal model with a simple structure can also provide references for the analysis of animal movement in nature and improve the energy efficiency of movement when analyzing energy in animal models. Partly, it simplifies the related research on animals and robots. In the future, researchers will further analyze and study the complex movement of animals combined with the biped robot model.

This study has certain restrictions. Caution should be exercised in promoting the results due to the small sample size, different test speed, various animal species, and ages. Sex differences in little quadrupeds are not significant. However, there are few studies on medium quadrupeds. Thus, an independent assessment is needed because the consistency of results cannot be guaranteed. Slope movement is a special case under complex terrain. The more complex terrains, including ice and snow-covered pavements, grasslands, debris, and others, will be discussed further in future research.

## 5. Conclusions

The findings indicated that, due to forelimbs being closer to the COM, during moving on the slope, the goat primarily regulated them. By lowering the initial included angle and change range, stability was preserved. In most cases, the forelimbs served as brakes, making the FP1 smaller than the FP2, and the hindlimbs drove the FP1 to be larger than the FP2. The forelimbs would not provide driving force until the slope angle was steep during uphill, which was accomplished by increasing the FP2 and impulse. Additionally, the hindlimbs exhibited the identical patterns of the FP1 and FP2 as the forelimbs. These findings contribute to the understanding of the kinetic adjustment strategies of quadruped limbs on special terrain.

## Figures and Tables

**Table 1:** Impulse values of the propulsive force and GRF during uphill and downhill.

Angle of Slope	Propulsive Force Impulse				Ground Reaction Force Impulse			
	Uphill		Downhill		Uphill		Downhill	
	Forelimb	Hindlimb	Forelimb	Hindlimb	Forelimb	Hindlimb	Forelimb	Hindlimb
10°	148.42 ± 0.54	126.00 ± 1.79	215.64 ± 1.61	67.97 ± 1.12	142.67 ± 0.70	98.57 ± 1.44	184.48 ± 1.61	57.39 ± 1.50
20°	144.12 ± 0.90	148.94 ± 1.14	177.62 ± 1.74	96.93 ± 1.59	138.52 ± 0.65	121.39 ± 1.94	155.24 ± 0.75	84.80 ± 0.78
30°	204.67 ± 1.37	89.18 ± 0.24	151.02 ± 1.43	99.93 ± 0.50	138.91 ± 1.09	76.35 ± 0.96	134.50 ± 1.16	90.83 ± 0.66

The impulse value in the table is the area value enclosed under the force-time curve.

## Data Availability

The data that support the findings of this study are available on reasonable request from the corresponding author. The data were not publicly available because of privacy or ethical restrictions.
